# Fluorescent peptide for detecting factor XIIIa activity and fibrin in whole blood clots forming under flow

**DOI:** 10.1016/j.rpth.2023.102291

**Published:** 2023-12-07

**Authors:** Yue Liu, Jennifer Crossen, Timothy J. Stalker, Scott L. Diamond

**Affiliations:** 1 Department of Chemical and Biomolecular Engineering, Institute for Medicine and Engineering, University of Pennsylvania, Philadelphia, Pennsylvania, USA; 2 Department of Medicine, The Cardeza Foundation for Hematologic Research, Thomas Jefferson University, Philadelphia, Pennsylvania, USA

**Keywords:** fibrin, thrombosis, alpha-2-antiplasmin, blood platelets

## Abstract

**Background:**

During clotting, thrombin generates fibrin monomers and activates plasma-derived transglutaminase factor (F) XIIIa; collagen and thrombin-activated platelets offer thrombin-independent cellular FXIIIa (cFXIIIa) for clotting. Detecting fibrin on collagen and tissue factor surfaces in whole blood clotting typically uses complex reagents like fluorescent fibrinogen or antifibrin antibody.

**Objectives:**

We want to test whether the peptide using the α2- antiplasmin crosslinking mechanism by FXIIIa is a useful tool in both monitoring FXIIIa activity, and visualize and monitor fibrin formation, deposition, and extent of crosslinking within fibrin structures in whole blood clots formed under flow.

**Methods:**

We tested a fluorescent peptide derived from α2-antiplasmin sequence (Ac-GNQEQVSPLTLLKWC-fluorescein) to monitor the location of transglutaminase activity and fibrin during whole blood clotting under microfluidic flow (wall shear rate, 100 s^−1^).

**Results:**

The peptide rapidly colocated with accumulating fibrin due to transglutaminase activity, confirmed by Phe-Pro-Arg-chloromethylketone inhibiting fibrin and peptide labeling. The FXIIIa inhibitor T101 had no effect on fibrin generation but ablated the labeling of fibrin by the peptide. Similarly, Gly-Pro-Arg-Pro abated fibrin formation and thus strongly attenuated the peptide signal. At arterial wall shear rate (1000 s^−1^), less fibrin was formed, and consequently, less peptide labeling of fibrin was detected compared with venous conditions. The addition of tissue plasminogen activator caused a reduction of both fibrin and peptide signals. Also, the peptide strongly colocalized with fibrin (but not platelets) in clots from laser-injured mouse cremaster arterioles. For clotting under flow, FXIIIa activity was most likely plasma-derived since a RhoA inhibitor did not block α2-antiplasmin fragment cross-linking to fibrin.

**Conclusion:**

Under flow, the majority of FXIIIa-dependent fibrin labeling with peptide during clotting was distal of thrombin activity. The synthetic peptide provided a strong and sustained labeling of fibrin as it formed under flow.

## Essentials

• Thrombin generates fibrin and activates factor XIIIa during clotting.
• Fluorescent peptide tracks transglutaminase in microfluidic blood clotting.
• Peptide rapidly colocalizes with fibrin due to transglutaminase *in vivo* and *in vitro.*
• For clotting under flow, FXIIIa activity was most likely plasma-derived.

## 1 Introduction

Factor (F) XIII is a transglutaminase enzyme that exists in the plasma and within the cytoplasm of hematopoietic cells [1,2], which usually comprises 2 pairs of subunits: A_2_ subunits that contain the active site and B_2_ subunits that act as carriers [3]. Cellular FXIIIa (cFXIIIa) is abundant within platelets, primarily in the form of a homodimer of FXIII-A, present in their cytoplasm [1], and small amount of the A_2_B_2_ form is also found in α-granules [1,4]. Platelets activated with both collagen and thrombin display cFXIIIa on their surface and release microparticles, a process requiring RhoA [5]. Platelet FXIII-A is important in modulating platelet phenotype [6,7], facilitating clot retraction by cross-linking extracellular fibrin and intracellular cytoskeletal proteins [8], and mediating extracellular cross-linking reactions [9].

Thrombin cleaves plasma-derived FXIII-A_2_B_2_ in the presence of Ca^2+^, causing the carrier B subunits to dissociate and expose the active site cysteine, which generates the active transglutaminase enzyme FXIIIa [10,11]. In the coagulation cascade, after fibrin polymerization, FXIIIa cross-links fibrin by forming covalent bonds at the γ and C-terminal α chains of fibrin [12], which plays a major role in stabilizing the clot when it forms. Activated FXIII cross-links fibrin, which affects the rheological properties of fibrin, leading to increased clot rigidity and higher resistance to mechanical stress [13,14]. FXIIIa also cross-links several inhibitors of fibrinolysis to fibrin, such as α2-antiplasmin (α2-AP) [15], thrombin activatable fibrinolysis inhibitor [16], and plasminogen activator inhibitor-2 [17]. These embedded proteins provide different roles in fibrin function. For example, α2-AP is a plasmin inhibitor and impedes fibrinolysis [18].

**Figure 1:**
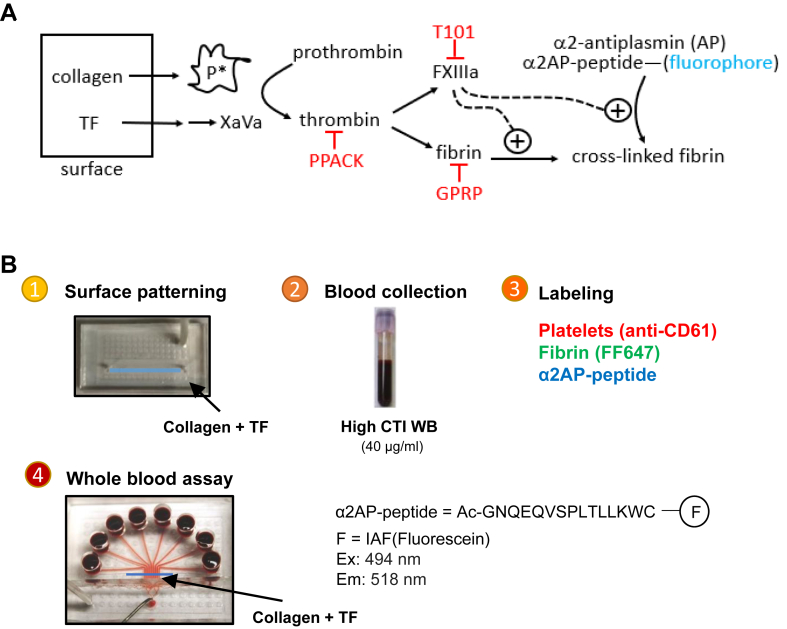
Schematic figure and microfluidics assay. (A) Schematic figure of high corn trypsin inhibitor WB flow over the collagen and tissue factor (TF) surface, generating activated platelet and factor Xa and Va, which activates prothrombin to form thrombin. Thrombin cleaves fibrin and activates factor XIIIa (FXIIIa), which cross-links fibrin, and α2-antiplasmin (AP) binds to the cross-linked fibrin. (B) In the microfluidic assay, collagen and tissue factor surfaces were patterned on glass slides using single-channel patterning devices. Then, high corn trypsin inhibitor WB was collected from healthy donors, platelets were labeled with anti-CD61, fibrin was labeled with AF647, and α2-APF was labeled. Actual flow assay was performed in 8-channel devices. CTI, corn trypsin inhibitor; GPRP, H-Gly-Pro-Arg-Pro-OH; PPACK, Phe-Pro-Arg-chloromethylketone; WB, whole blood.

In this study, we explored a method to study fibrin formation and cross-linking by FXIIIa during clotting. Tung et al. [19,20] developed a fluorescently labeled peptide fragment derived from α2-AP (N_13_QEQVSPLTLLK_24_) that contains the same glutamine residue that is cross-linked by FXIIIa to bind α2-AP to fibrin. Their study demonstrated that the peptide attaches to fibrin, specifically via FXIIIa cross-linking [19,20]. We hypothesized that a peptide utilizing the α2-AP cross-linking mechanism by FXIIIa would be a useful tool in both monitoring FXIIIa activity as well as visualizing and monitoring fibrin formation, deposition, and extent of cross-linking within fibrin structures in whole blood clots formed under defined flow conditions (Figure 1A).

Previous research on the functions of α2-AP cross-linked to fibrin has used various mice models [18,19,21] or human blood in the stagnant [21] and arterial shear condition only [22]. Using our previously developed 8-channel microfluidic assay (Figure 1B), we here formed whole blood clots under flow in the presence of α2-AP fragment (α2-APF), monitoring for α2-APF deposition, its colocalization with fibrin, and activity of FXIIIa over time with rapid change in conditions [23], fluids, or flowrate (both venous and arterial conditions) [24]. Confocal microscopy was also used to validate the results of the *in vitro* microfluidic assay. Results obtained in a laser-injury cremaster arteriole model were consistent with observations from human blood clotting under microfluidic flow.

## 2 Methods

### 2.1 Materials

The following reagents were obtained: Sigmacote (Millipore Sigma, Cat# SL2-100ML), collagen (type I; Chrono-Log, Cat# 385), Dade Innovin prothrombin time reagent (Siemens, Cat# B4212-40), corn trypsin inhibitor (CTI; Haematologic Technologies, Cat# CTI-01), fluorescently labeled fragment derived from α2-AP (Ac-GNQEQVSPLTLLKW-C[IAF], α2-APF, CPC Scientific), antihuman CD61 antibody (BD Biosciences, Cat# 555754), Alexa Fluor 647 Conjugated Human Fibrinogen (Life Technologies, Cat# F35200), H-Gly-Pro-Arg-Pro-OH (GPRP; Millipore Sigma, Cat# 03-34-0001), Phe-Pro-Arg-chloromethylketone (PPACK; Haematologic Technologies, Cat# FPRCK-01), 1,3,4,5-tetramethyl-2-([2-oxopropyl]thio) imidazolium chloride (T101; Zedria GmbH), Recombinant Human Tissue Plasminogen Activator Protein (tPA; Abcam, Cat# ab92637), and rhosin (Rho inhibitor; Millipore Sigma, CAS 1173671-63-0).

### 2.2 Preparation and characterization of collagen/tissue factor surface

First, glass slides were washed with ethanol and deionized water, followed by drying with filtered air. Subsequently, sigmacote was applied to create a hydrophobic surface. Then, 5 μL of fibrillar collagen was perfused through a patterning channel (250 μm wide × 60 μm high) of the microfluidic device to create a single 250 μm-wide stripe of fibrillar collagen for the experiment. Next, the collagen was rinsed, and lipidated tissue factor (TF) was absorbed to the collagen surface via the perfusion of 5 μL of Dade Innovin prothrombin time reagent (20 nM stock concentration), followed by incubation for 30 minutes without flow and subsequently rinsed and blocked with 0.5% bovine serum albumin (BSA, 20 μL) [23,25,26].

### 2.3 Blood collection and preparation

Blood was drawn through venipuncture into a syringe containing a high concentration of CTI (40 μg/mL) from healthy donors who self-reported being free of alcohol use for at least 72 hours and medication for at least a week prior to blood collection. Consent was provided by all donors under approval of the University of Pennsylvania’s Institutional Review Board. Blood was treated with antihuman CD61 antibody (1:50 v/v in whole blood) and Alexa Fluor-conjugated human fibrinogen (1.5 mg/mL stock solution, 1:80 v/v in whole blood) for platelets and fibrin labeling. CD61 binds both platelets and white blood cells; however, the deposits on collagen under flow contained almost no white blood cells until much later. Even at low resolution, any white blood cells present would be discovered morphologically. α2-APF was added to the collected whole blood at a final concentration of 5 μM, and all experiments started within 5 minutes of the phlebotomy. For each set of experiments, blood samples from N ≥ 2 donors were taken with 1 clot formed per channel.

### 2.4 Microfluidic clotting assay on collagen surfaces with or without tissue factor

A polydimethylsiloxane flow device with 8 channels was sealed perpendicularly to collagen/TF surfaces, creating 8 parallel prothrombotic patches (250 × 250 μm) as previously reported [25,26]. Depending on the experiment duration, the channel height was either 60 μm or 120 μm in order to prevent occlusion [27] while keeping the shear rate at venous (100 s^−1^) or arterial level (1000 s^−1^). Treated blood was perfused across the 8 channels by withdrawal through a single outlet. Drug-treated blood (whole blood with reagents such as tPA) was added to the inlet reservoir without interrupting the flow, thus providing a quick change in perfusion pharmacology in less than 15 seconds without affecting the hemodynamics between channels during the perfusion switch. The initiation of clotting events was conducted simultaneously in a microfluidic device controlled by a syringe pump to regulate the initial wall shear rate. IX81 epifluorescence microscopy (Olympus America Inc) at 10 × magnification was used to monitor platelet activity. Blood from at least 2 donors was used for each experiment, and the total platelet clot mass was measured by fluorescence intensity. Bright-field imaging was not used due to the variation in thickness of the flowing blood above the clot over time. Images were captured using a charged coupled device camera (Hamamatsu) and analyzed with ImageJ software. In order to avoid side-wall effects, only the central 75% of the channel was taken into account.

### 2.5 Confocal microscopy

To determine the 3-dimensional orientation of platelets, fibrin, and α2-APF, we used confocal microscopy to generate images of clots in the microfluidic device. Initially, a monolayer of clot was formed under high CTI whole blood (WB) perfused over collagen/TF (100 s^−1^) for 90 seconds, followed by buffer (HEPES buffer solution) containing α2-AP peptide for 200 seconds. Then, BSA (+5 mM CaCl_2_) was perfused for 2 minutes to remove any remaining blood in the channels, followed by 4% paraformaldehyde (+5mM CaCl_2_) to replace any remaining BSA (+5 mM CaCl_2_) in the wells and perfused for 2 minutes to fix the clots. After the 2-minute perfusion with 4% paraformaldehyde (+5 mM CaCl_2_), the device was transferred to a confocal microscope with the clots still maintained in the 8-channel microfluidic device. Z-stack images were taken of fixed clots using the Leica TCS SP8 laser scanning confocal microscope at the Cell & Developmental Biology Microscopy Core at the University of Pennsylvania.

### 2.6 Response to vascular injury in mouse cremaster arterioles

Animal studies were approved by the Institutional Animal Care and Use Committee of Thomas Jefferson University. Male C57Bl/6 mice were anesthetized using a ketamine/xylazine/acepromazine (100/10/2 mg/kg) cocktail and prepared for intravital microscopy to visualize the cremaster muscle microcirculation as previously described [28]. Mice were infused with fluorescently labeled antibodies against CD41 (clone MWReg30, F[ab]_2_, 0.12 μg/g body weight) and fibrin (clone 59D8, 0.2 μg/g body weight). The α2-AP peptide (5 nmol in 100 μL normal saline) was infused via a cannula placed in the jugular vein. The intravital imaging system comprised a Zeiss AxioExaminer.D1 upright microscope with a 20× (1.0 numerical aperture water immersion objective coupled to a Yokogawa CSU-X1 spinning disk confocal scanner. Diode-pumped solid-state lasers (488 nm, 568 nm, and 640 nm) with acousto-optic tunable filter control were used as the fluorescence excitation light source (LaserStack, Intelligent Imaging Innovations). Images were acquired using a Hamamatsu Orca-Flash sCMOS digital camera (Bridgewater). Laser-induced vascular injuries were made using an Ablate! ablation laser system that is fired through the microscope objective and focused on the specimen (Intelligent Imaging Innovations). All components were controlled and synchronized using Slidebook 6.0 image acquisition and analysis software (Intelligent Imaging Innovations).

### 2.7 Statistical analysis

Data and images were analyzed in GraphPad Prism v10.0.2.232 and ImageJ, respectively. Statistical analysis was performed using the Student’s *t*-test to compare different conditions to the control. A *P* < .05 was considered significant.

## 3 Results

### 3.1 α2-antiplasmin fragment colocalizes with fibrin and deposits from the initiation of clotting

**Figure 2:**
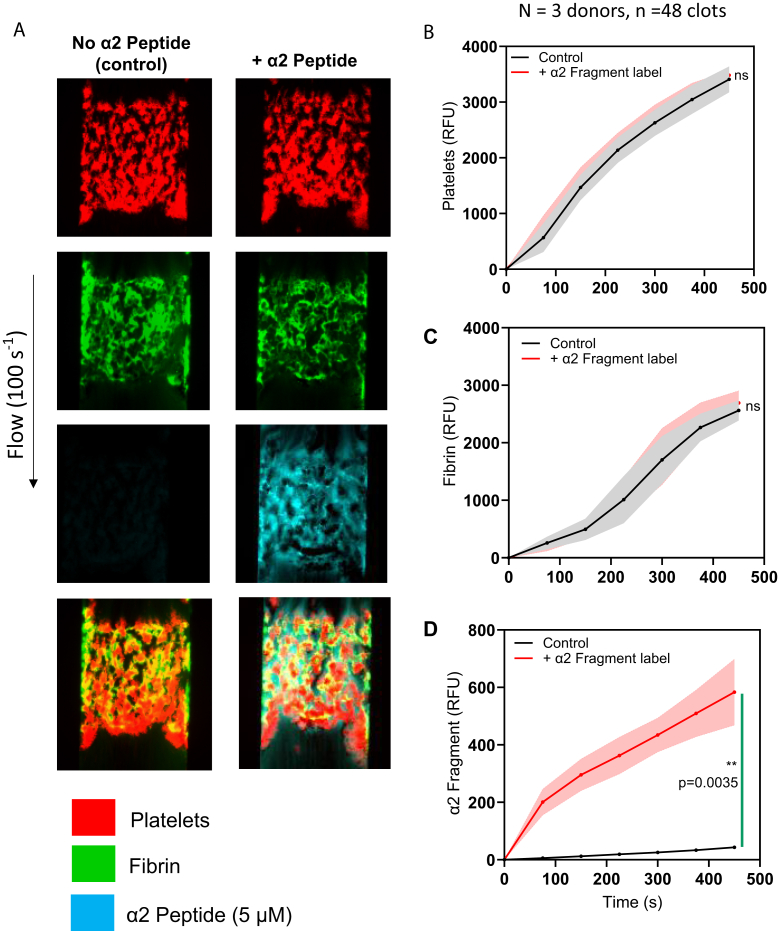
α2-antiplasmin fragment (APF) colocalizes with fibrin and deposits from the initiation of clotting. Whole blood clots were formed under venous shear (100 s^−1^) and monitored for platelet deposition, fibrin formation, and α2-APF deposition. (A) Representative images of clots formed at ±5 μM α2-APF. (B) Platelet deposition of clots formed at ±5 μM α2-APF. (C) Fibrin formation of clots formed at ±5 μM α2-APF. (D) α2-APF deposition of clots formed at ±5 μM α2-APF. ∗*P* < .05; ∗∗*P* < .005, ∗∗∗*P* < .0005, ns, not significant.

Using the microfluidic assay (Figure 1B), we perfused human whole blood at venous shear conditions (100 s^−1^) in the presence of α2-APF, monitoring for α2-APF accumulation, colocalization with fibrin, and activity of FXIIIa over time. In Figure 2A, we first established the assay and compared the formation and morphology of clots formed in the presence and absence of the α2-APF. We observed similar fibrin formation and platelet deposition for clots formed with and without α2-APF conditions, and the platelet and fibrin’s fluorescent intensity (FI) for these 2 conditions were identical (Figure 2B, C), indicating no significant impact on clot formation due to the presence of α2-APF. In Figure 2A, there was colocalization of fibrin formation (green, second panel) and α2-APF (cyan, third panel from top), which would be expected for α2-APF cross-linking to fibrin by FXIIIa. Figure 2D shows the FI for α2-APF.

### 3.2 Specificity of the α2-antiplasmin fragment to fibrin and factor XIIIa activity

**Figure 3:**
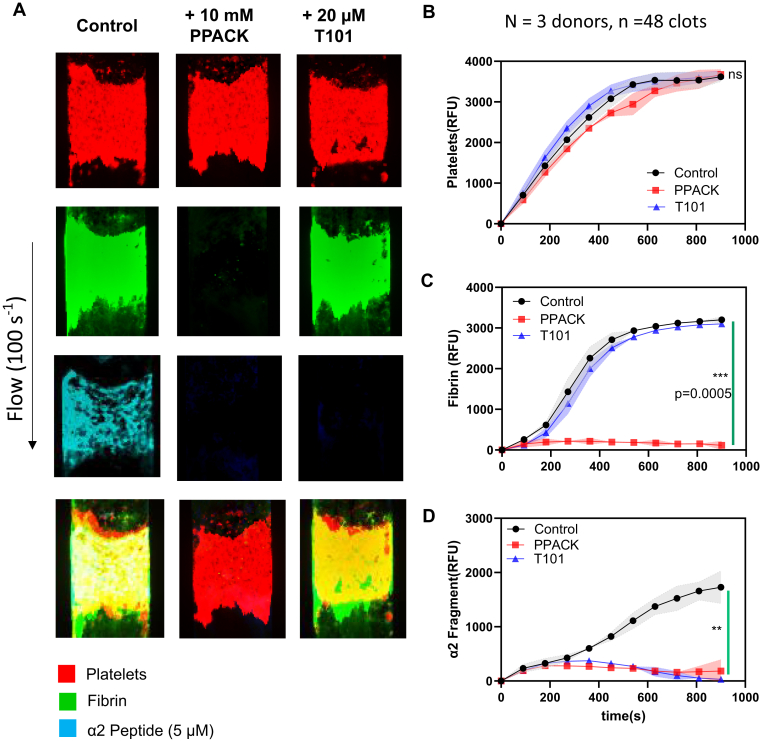
Specificity of the α2-antiplasmin fragment (APF) to fibrin and factor XIII formation. High corn trypsin inhibitor whole blood clots were formed at venous shear (100 s^−1^) for 900 seconds (15 minutes) with ±10 mM Phe-Pro-Arg-chloromethylketone (PPACK) (no thrombin) or ±20 μM T101 (no FXIIIa) and monitored for platelet deposition, fibrin formation, and α2-APF deposition. (A) Representative images of platelet (red, top row), fibrin (green, second row), and α2-APF (cyan, third row) for all conditions. (B) Platelet accumulation for control (black), +10 mM PPACK (red), and +20 μM T101 (blue). (C) Fibrin accumulation for all conditions. (D) α2-APF accumulation for all conditions. ∗*P* < .05; ∗∗*P* < .005, ∗∗∗*P* < .0005, ns, not significant.

We investigated the requirements for α2-APF labeling of fibrin and FXIIIa formation. In Figure 3, we formed clots with 10 mM PPACK to inhibit thrombin (thus no fibrin polymerization and no plasma FXIIIa activation) and 20 μM T101 to inhibit FXIIIa (no fibrin cross-linking of α2-APF to fibrin) at venous shear conditions (100 s^−1^). We monitored the accumulation of α2-APF to clots over time to determine if there were nonspecific interactions with α2-APF and other clot components. Overall, we observed no substantial difference for platelet FI (Figure 3B). The addition of T101 did not influence fibrin FI (Figure 3C); however, PPACK blocked fibrin generation (Figure 3A, C), as expected. In the presence of PPACK, little fibrin was generated, and concomitantly little α2-APF was incorporated into the clot (Figure 3D). In the presence of FXIIIa inhibitor T101 under flow conditions, platelets accumulating on collagen and generating thrombin did not result in sufficient transglutaminase activity (cFXIIIa) to allow for the detection of α2-APF accumulation within the platelet/fibrin clot (Figure 3A, right, D).

### 3.3 Specificity of the α2-antiplasmin fragment to fibrin rather than thrombin

**Figure 4:**
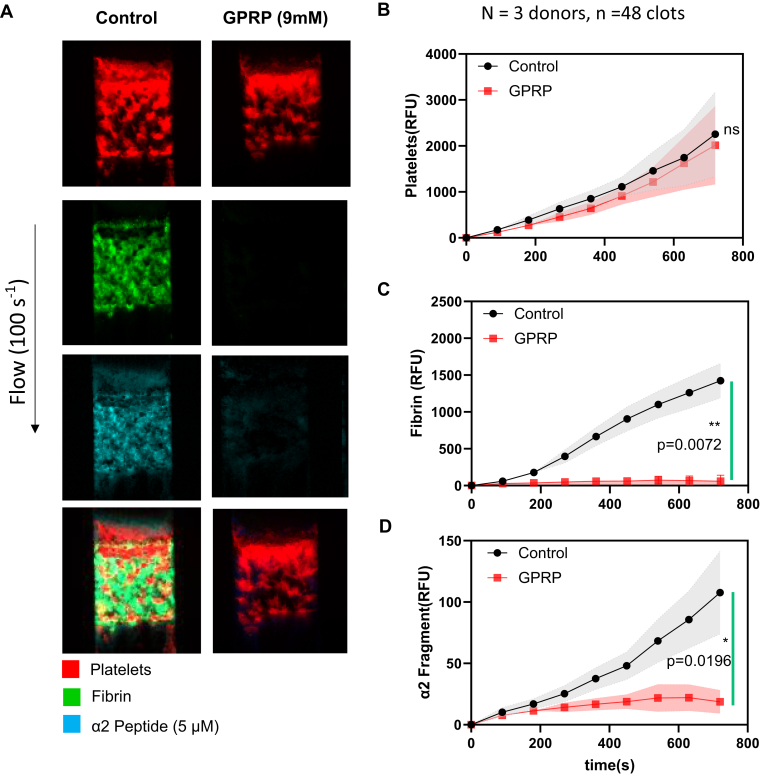
Specificity of the α2-antiplasmin fragment (APF) to fibrin rather than thrombin. High corn trypsin inhibitor whole blood clots were formed at venous shear (100 s^−1^) for 720 seconds with ±9 mM H-Gly-Pro-Arg-Pro-OH (GPRP) and monitored for platelet deposition, fibrin formation, and α2-APF deposition. (A) Representative images of platelet (red, top row), fibrin (green, second row), and α2-APF (cyan, third row) for all conditions. (B) Platelet accumulation for control (black) and +9 mM GPRP (red). (C) Fibrin accumulation for all conditions. (D) α2-APF accumulation for all conditions. ∗*P* < .05; ∗∗*P* < .005, ∗∗∗*P* < .0005, ns, not significant.

To investigate the localization of the peptide to fibrin in the presence of thrombin, 9 mM of GPRP, which suppresses the early steps of fibrin polymerization, was added to prevent fibrin polymerization. High corn trypsin inhibitor (HCTI)-treated whole blood was perfused over collagen and TF surface at venous shear conditions (100 s^−1^) in the presence of α2-APF, with or without GPRP, monitoring for platelet deposition, fibrin deposition, and α2-APF deposition over time. In Figure 4A, we first established the assay and compared the formation and morphology of clots formed in the presence and absence of the GPRP. We observed similar platelet deposition for clots formed with and without GPRP (red, first panel). Fibrin was absent in the GPRP condition, as expected (green, second panel), and very little α2-APF accumulated, even though thrombin can be generated in this condition. The platelet FI for these 2 conditions was almost similar (Figure 4B), indicating no significant impact on platelet deposition due to the absence of fibrin. There was no fibrin formation in the presence of GPRP since GPRP blocked fibrin polymerization (Figure 4C). The FI for α2-APF in the control condition was about 6-fold greater than that of the GPRP condition (Figure 4D).

### 3.4 T101 dose-dependently inhibit α2-antiplasmin fragment deposition under flow

**Figure 5:**
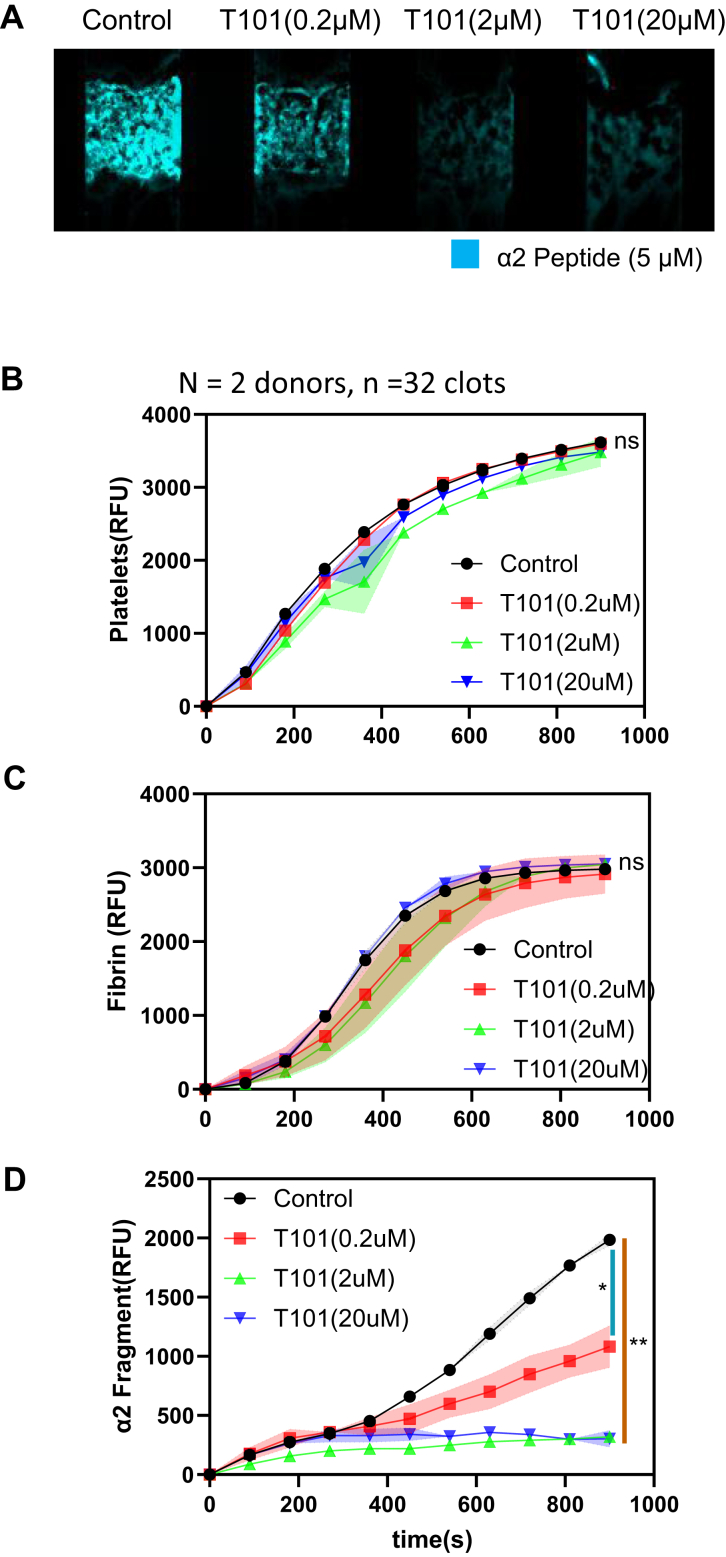
T101 dose response with α2-antiplasmin fragment (APF). High corn trypsin inhibitor whole blood clots were formed at venous shear (100 s^−1^) for 900 seconds ± different concentrations of T101 (no factor XIIIa) and monitored for platelet deposition, fibrin formation, and α2-APF deposition. (A) Representative images of α2-APF. (B) Platelet fluorescent intensity (FI) for control conditions and conditions with different concentrations of T101. (C) Fibrin FI for all conditions. (D) α2-APF FI for all conditions. ∗*P* < .05; ∗∗*P* < .005, ∗∗∗*P* < .0005, ns, not significant.

To investigate whether the concentration of T101 can produce a dose-dependent inhibition of α2-APF deposition, different concentrations of T101 were tested using the microfluidic device. We performed a T101 dose-response experiment at venous shear conditions (100 s^−1^) over collagen/TF surfaces. As the concentration of T101 increased, less α2-APF was incorporated into the clot (Figure 5A). Overall, there was little effect of T101 on platelet FI at the different concentrations of T101 (Figure 5B). Addition of T101 did not influence fibrin FI (Figure 5C). As the concentration of T101 was increased from 0 to 2 μM, the FI for α2-APF decreased. The IC50 for T101 was about 0.2 μM under flow conditions (Figure 5D), consistent with earlier reports of T101 potency [5].

### 3.5 Less fibrin formation and α2-antiplasmin fragment deposition under arterial condition

**Figure 6:**
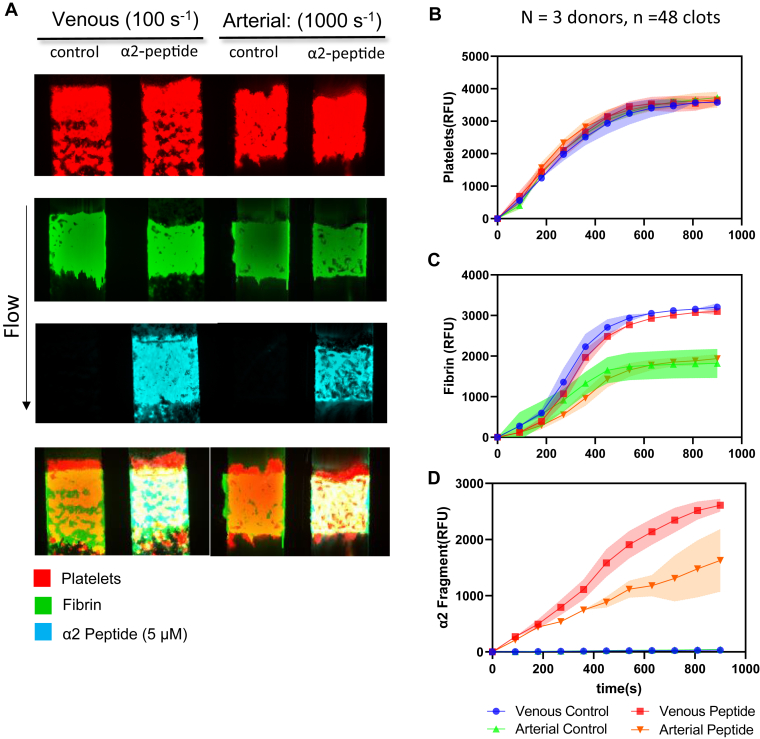
High corn trypsin inhibitor whole blood with or without α2-antiplasmin fragment (APF) under venous and arterial conditions. High corn trypsin inhibitor whole blood clots were formed at venous shear (100 s^−1^) and arterial shear (1000 s^−1^) for 900 seconds ± α2-APF and monitored for platelet deposition, fibrin formation, and α2-APF deposition. (A) Representative images of platelet (red, top row), fibrin (green, second row), and α2-APF (cyan, third row) for all conditions. (B) Platelet fluorescent intensity (FI) for all conditions. (C) Fibrin FI for all conditions. (D) α2-APF FI for all conditions.

To investigate the effect of flow rate on α2-APF deposition, we performed experiments under both venous (100 s^−1^) and arterial (1000 s^−1^) conditions with or without α2-APF. Accumulation of platelets was not altered by the presence of α2-APF under either venous or arterial flow conditions (Figure 6A, top, B). In Figure 6A, C, there was ∼50% reduction in fibrin formation under arterial conditions compared with the venous flow condition. At either flow condition, the fibrin FI was similar in the presence or absence of α2-APF (Figure 6C). The α2-APF FI under the venous condition was about 1.6 times higher than the arterial condition (Figure 6D), consistent with a higher amount of fibrin generated at the lower shear condition. Overall, less fibrin was made under the arterial condition, and less α2-APF was incorporated into the clot despite similar levels of platelets under all conditions.

### 3.6 α2-antiplasmin fragment and lytic susceptibility of fibrin

**Figure 7:**
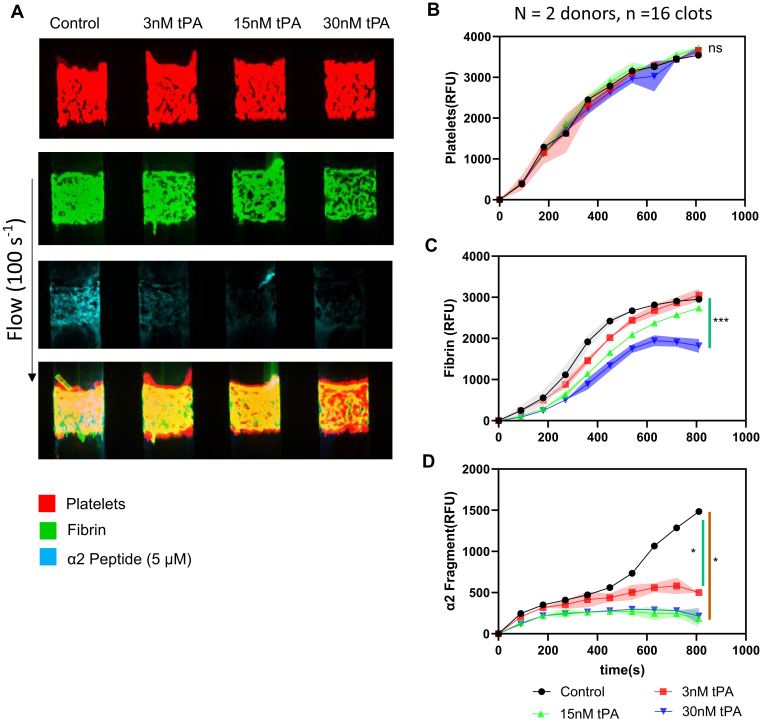
Tissue plasminogen activator (tPA) dose response with α2-antiplasmin fragment (APF). High corn trypsin inhibitor whole blood clots were formed at venous shear (100 s^−1^) for 900 seconds with different concentrations of tPA (0 nM, 3 nM, 15 nM, and 30 nM) and monitored for platelet deposition, fibrin formation, and α2-APF deposition. (A) Representative images of platelet (red, top row), fibrin (green, second row), and α2-APF (cyan, third row) for all conditions. (B) Platelet fluorescent intensity (FI) for all conditions. (C) Fibrin FI for all conditions. (D) α2-APF FI for all conditions. ∗*P* < .05; ∗∗*P* < .005, ∗∗∗*P* < .0005, ns, not significant.

We added tPA into WB to establish if there was an effect of α2-APF cross-linking to fibrin, potentially via outcompeting α2-AP cross-linking to fibrin. We perfused CTI-treated WB with different concentrations of tPA (3 nM, 15 nM, and 30 nM) over collagen/TF at 100 s^−1^ for 15 minutes. The accumulation of platelet, fibrin, and α2-APF was observed over time. In Figure 7A, we observed similar platelet deposition for clots formed with different tPA concentrations (red, first panel). As we increased the tPA concentration, we observed less fibrin accumulation (green, second panel) and less α2-APF incorporation (cyan, third panel) under flow. The platelet FI for these 4 conditions was largely unchanged by the presence of tPA during clotting (Figure 7B). When more tPA was added to WB, more fibrin was dissolved (Figure 7C). As the concentration of tPA increased from 0 nM to 15 nM, the α2-APF FI decreased, but when we increased the concentration from 15 nM to 30 nM, the FI of the α2-APF did not decrease further (Figure 7D). In Figure 7D, the fibrin labeling by cross-linking with α2-APF did not appear to alter the lytic susceptibility of the clot to tPA.

**Figure 8:**
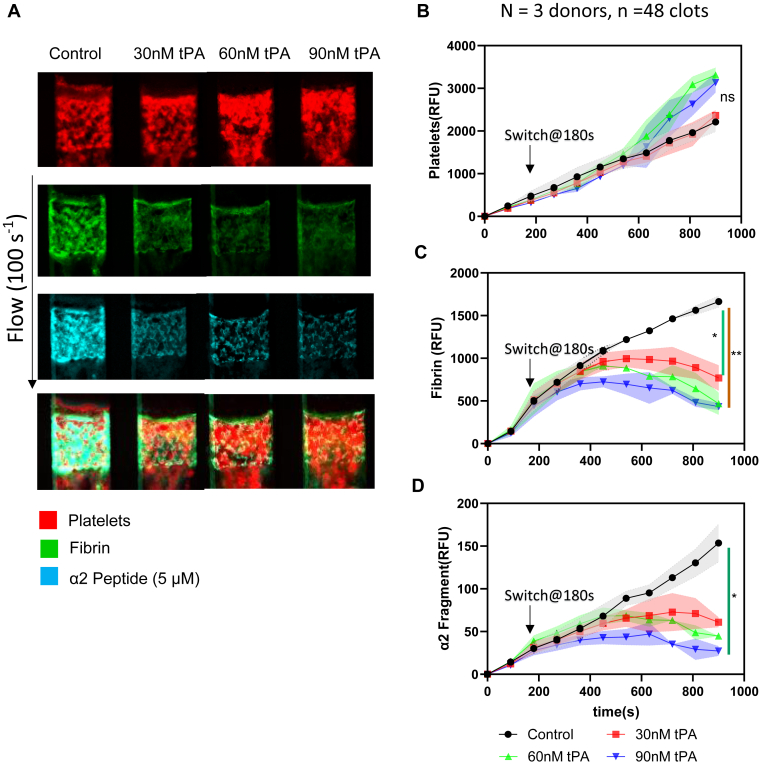
Switching experiments with different concentrations of tissue plasminogen activator (tPA). Control condition High corn trypsin inhibitor whole blood clots were formed at venous shear (100 s^−1^) for 180 seconds, then switched to high corn trypsin inhibitor WB with different concentrations of tPA (0 nM, 30 nM, 60 nM, and 9 0nM) and monitored for platelet deposition, fibrin formation, and α2-antiplasmin fragment (APF) deposition. (A) Representative images of platelet (red, top row), fibrin (green, second row), and α2-APF (cyan, third row) for all conditions. (B) Platelet fluorescent intensity (FI) for all conditions. (C) Fibrin FI for all conditions. (D) α2-APF FI for all conditions. ∗*P* < .05; ∗∗*P* < .005, ∗∗∗*P* < .0005, ns, not significant.

We next evaluated the effect of switching from the control WB condition to WB with tPA at 180 seconds. We perfused CTI-treated WB for 180 seconds over collagen/TF at 100 s^−1^, then switched to HCTI-treated WB with different tPA concentrations (30 nM, 60 nM, and 90 nM) for 720 seconds. The accumulation of platelet, fibrin, and α2-APF was observed over time. In Figure 8A, similar platelet deposition for clots was formed (red, first panel). As the tPA concentration increased in the switched WB, less net fibrin was generated (green, second panel) and less α2-APF was deposited (cyan, third panel) under flow. The platelet FI for these 4 conditions was about the same (Figure 8B), indicating that tPA had no significant impact on platelet deposition. When more tPA was added into the switched WB, less fibrin was observed due to the increased lytic state (Figure 8C). A higher tPA concentration in the switched WB resulted in lower levels of α2-APF FI (Figure 8D), as expected for elevated fibrin lysis.

### 3.7 Confocal microscopy: α2-antiplasmin fragment was localized to fibrin on the exterior of clots, while the interior of dense platelet deposits lacked substantial α2-antiplasmin fragment labeling

**Figure 9:**
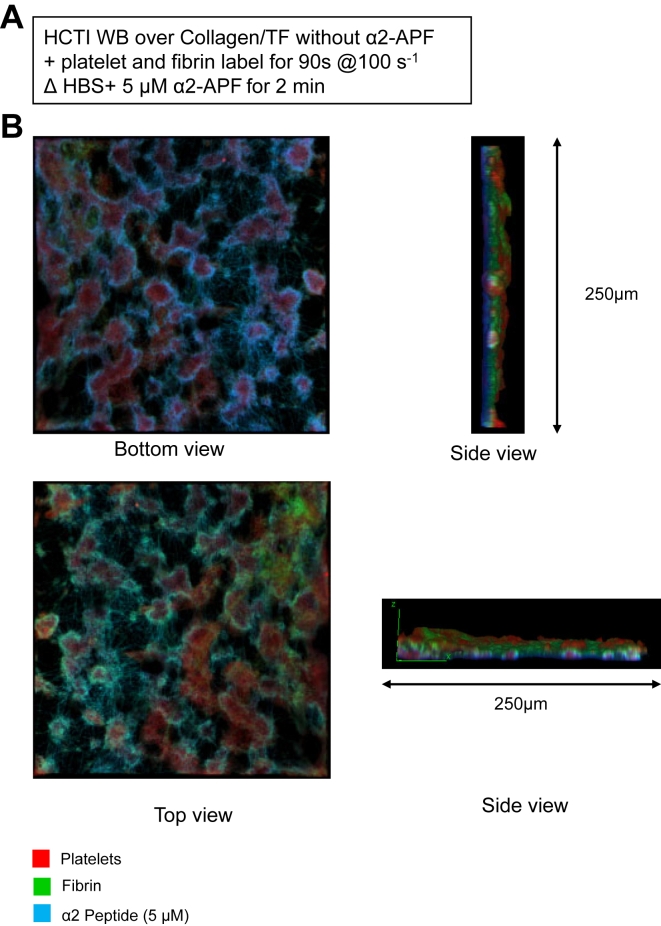
Confocal images for clots with α2-antiplasmin (AP) peptide. (A) High corn trypsin inhibitor whole blood monolayers were formed at venous shear (100 s^−1^) for 90 seconds with platelet and fibrin label, then washed with buffer (HEPES buffer solution) added α2-AP peptide for 2 minutes. (B) Confocal image labeled with platelet, fibrin, and α2-AP fragment (α2-APF) with top, bottom, and side views.

We perfused HCTI WB with platelet and fibrin labels at venous shear conditions (100 s^−1^) over collagen/TF surfaces for 90 seconds to form a thin clot. Clots were then washed with buffer (HEPES buffer solution) with 5 μM α2-APF for 2 minutes (Figure 9A). Confocal images for clots with labeling platelet, fibrin, and α2-APF are shown from the bottom, top, and side views (Figure 9B, Supplementary Movie S1). These images illustrate that α2-APF predominantly colocalized near the fibrin surrounding the dense platelet aggregates, while platelets were distributed on the collagen/TF surface as dense aggregates lacking α2-APF staining. Interestingly, little α2-APF was detected at the top layer of the clot exposed to flow.

### 3.8 α2-antiplasmin fragment colocalizes with fibrin during thrombus formation *in vivo*

**Figure 10:**
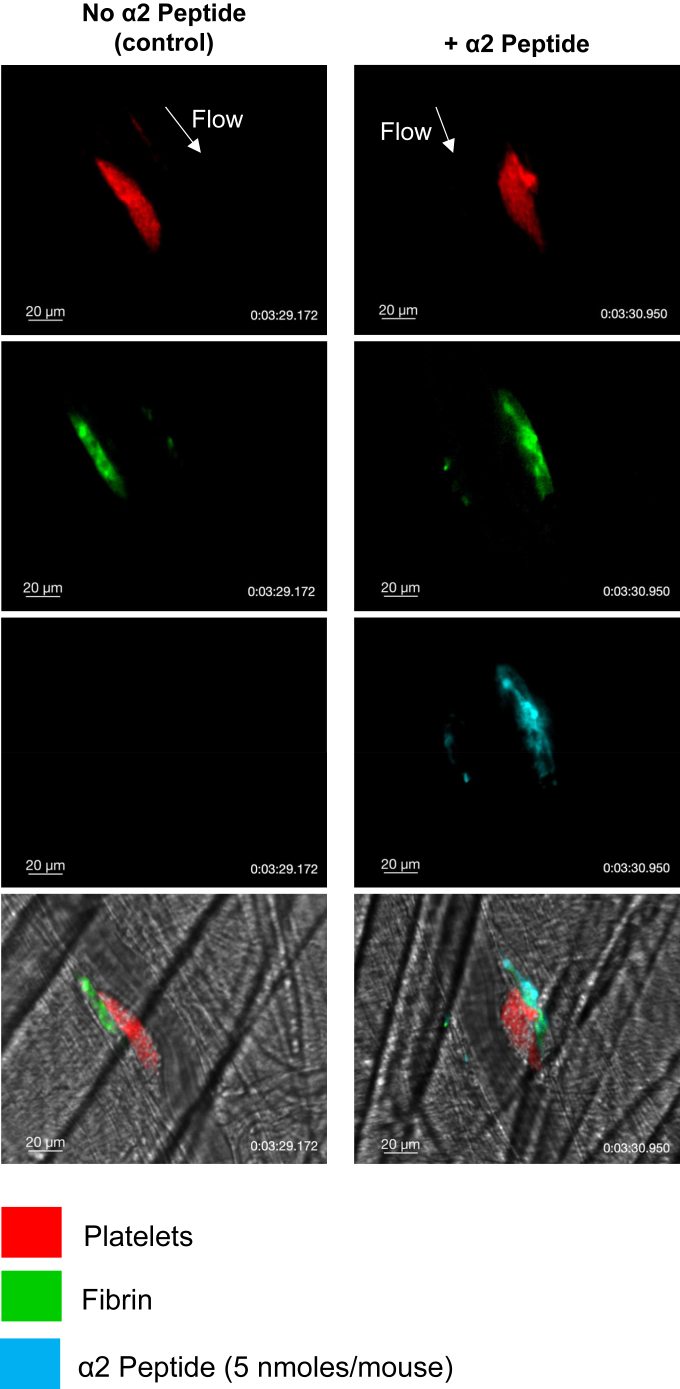
α2-antiplasmin fragment colocalizes with fibrin during thrombus formation *in vivo*. Representative images of thrombi 3.5 minutes after laser-induced vascular injury of mouse cremaster arterioles. Mice were infused with vehicle (saline, left column) or the α2-antiplasmin fluorescent peptide (right column). White arrows indicate the direction of flow. The merged fluorescence image is overlaid on the bright-field background. Images are representative of 18 thrombi from 3 mice.

We tested the colocalization of α2-APF by penetrating injuries to cremaster muscle arterioles in adult C57Bl/6 mice. Mice were infused with fluorescently labeled platelets, fibrinogen, and α2-APF. We took confocal images for conditions with or without α2-APF after laser-induced vascular injuries (Figure 10, Supplementary Movie S2). We observed similar platelet deposition for clots formed with or without α2-APF (red, first panel). There was colocalization of fibrin formation (green, second panel) and α2-APF (cyan, third panel), which was consistent with the *in vitro* microfluidic experiments using human blood. In contrast to fibrin/α2-APF colocalization, the dense platelet mass (red) lacked substantial incorporation of α2-APF.

### 3.9 Most α2-antiplasmin tag is likely due to plasma-derived factor XIIIa

**Figure 11:**
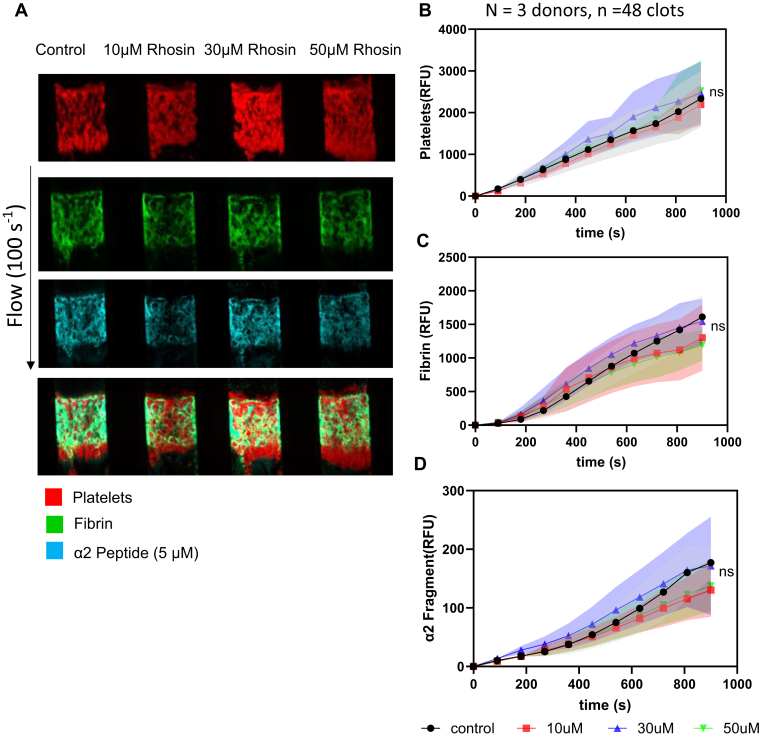
Rhosin dose response with α2-antiplasmin fragment (APF). High corn trypsin inhibitor whole blood clots were formed at venous shear (100 s^−1^) for 900 seconds with different concentrations of rhosin (0 μM, 10 μM, 30 μM, and 50 μM) and monitored for platelet deposition, fibrin formation, and α2-APF deposition. (A) Representative images of platelet (red, top row), fibrin (green, second row), and α2-APF (cyan, third row) for all conditions. (B) Platelet fluorescent intensity (FI) for all conditions. (C) Fibrin FI for all conditions. (D) α2-APF FI for all conditions. Ns, not significant.

To investigate whether the α2-AP tag is due to plasma-derived FXIIIa or cFXIIIa, we performed experiments with HCTI WB and added different concentrations of rhosin (0 μM, 10 μM, 30 μM, and 50 μM), a RhoA inhibitor, which significantly decreased cFXIII translocation, at venous shear condition (100 s^−1^) for 15 minutes. The accumulation of platelet, fibrin, and α2-APF was observed over time. In Figure 11A, we observed similar platelet deposition (red, first panel), fibrin formation (green, second panel), and α2-APF accumulation (cyan, third panel) for clots formed with different rhosin concentrations. The platelet FI for these 4 conditions was largely unchanged by the presence of rhosin during clotting (Figure 11B). The addition of rhosin did not influence fibrin and α2-APF FI (Figure 11C, D).

## 4 Discussion

We performed a series of *in vitro* experiments with human blood using α2-APF to study fibrin formation and the locality of cross-linking activity by FXIIIa during whole blood clotting under flow. This peptide sequence is well recognized to serve as a substrate and be cross-linked to fibrin by the transglutaminase activity of plasma FXIIIa or cFXIIIa [5].

Under flow conditions, α2-APF colocalized with fibrin from the initiation of clotting at t = 0. To confirm the specificity α2-APF for fibrin labeling, we used inhibitors of thrombin and FXIIIa. Both PPACK and T101 ablated the α2-APF signal compared with the control condition, which was 10 times greater than the PPACK or T101 condition. The requirement for transglutaminase activity for fibrin labeling by α2-APF is strongly supported by the T101 result. PPACK creates an experimental condition where (1) no thrombin activity is present, (2) no fibrin is formed, (3) no plasma FXIII is activated, and (4) few coated platelets are generated, which in turn prevents cFXIIIa presentation on membranes. The use of GPRP creates a fibrin-free environment in the experiment where (1) thrombin is present, (2) coated platelets can form on collagen in the presence of thrombin (via the extrinsic pathway) and present cFXIIIa, (3) and plasma FXIIIa can be generated by thrombin. The α2-APF signal on fibrin without GPRP was about 6 times that of the condition with GPRP. We also used confocal images for clots to illustrate that α2-APF predominantly localized near the fibrin. The lack of α2-APF labeling within the dense platelet aggregates may be a result of poor α2-APF entry or lack of coated platelets in the interior of the platelet mass due to the sorting of coated platelets to the exterior of the platelet mass [29]. A similar lack of α2-APF labeling of platelet mass was observed *in vivo* with adult C57Bl/6 mice, where the hemostatic platelet mass covering the laser injury lacked α2-APF labeling, while the fibrin that was formed at the injured vessel wall and outside the vessel was highly labeled with α2-APF.

We also studied the relationship between α2-APF and the lytic susceptibility of fibrin by the addition of tPA. As we increased the concentration of tPA, there was less fibrin and concomitantly less α2-APF deposition under flow. Similar results were obtained in the switching experiment where tPA was added after 180 seconds of clotting. Competitions may exist, as shown in Figure 7. The small peptide may have a kinetic advantage over antiplasmin cross-linking, thus making the fibrin more susceptible to lysis. Alternatively, peptide-labeled fibrin may interfere with plasminogen activation or fibrinolysis. The peptide may not be a fully reliable metric of fibrin generation since fibrin both forms and dissolves in the presence of tPA.

Recently, Mitchell et al. [30] evaluated the role of platelet-derived FXIII-A in platelet function. In their study, platelet rich plasma and washed platelets were predominantly used under nonflow conditions with exogenously added thrombin. They found a role for platelet FXIII-A during whole blood clotting under flow on fibrinogen but not collagen. In our study, we used whole blood perfusion and surface presentation of TF to drive endogenous thrombin, fibrin generation, and fibrin-associated FXIIIa cross-linking activity. To investigate if the peptide sequence is cross-linked to fibrin by the transglutaminase activity of plasma FXIIIa or cFXIIIa, experiments with different concentrations of rhosin were performed at a venous shear rate of 100 s^−1^, a condition that optimizes the generation of fibrin and the detection of fibrin-localized FXIIIa cross-linking activity. Rhosin is a RhoA inhibitor, and RhoA mediates Ca^2+^-independent pathways in the surface exposure of cFXIII during platelet activation, specifically induced by collagen and protease-activated receptors [5]. Since the RhoA inhibitor did not affect the labeling of fibrin, we conclude that FXIIIa activity was plasma-derived in experiments of thrombosis on collagen/TF surfaces under flow.

While not having information on the race/ethnicity of participants in a table is a limitation of our study, it should be noted that the impact is negligible. While including this data would have provided a more nuanced understanding of socio-cultural determinants of health, the primary focus of our research remains on monitoring transglutaminase activity and fibrin formation during whole blood clotting under microfluidic flow. It is important to recognize that the absence of this information does not undermine the validity of our core findings.

In conclusion, the detection of fibrin in flow assays typically requires delicate and expensive reagents such as fluorescent antifibrin or fibrinogen. The use of a synthetic peptide is far easier than antifibrin or fluorescent fibrinogen for point-of-care devices that contain reagents presupplied on disposable microfluidic chips. Using the antiplasmin sequence as an FXIIIa substrate during whole blood clotting under flow, we demonstrate fibrin labeling requires thrombin, fibrin, and FXIIIa activity.
