# Supplementary material for: Fluorescent peptide for detecting factor XIIIa activity and fibrin in whole blood clots forming under flow
Source: Res Pract Thromb Haemost. 2023 Dec 7;8(1):102291. doi: 10.1016/j.rpth.2023.102291 (PMC10787300; doi:10.1016/j.rpth.2023.102291)
Supplement: Supplementary Material [file mmc3.docx]

**Supplement**

**Synthetic peptide for real time fluorescence detection of FXIIIa activity and fibrin in whole blood clots forming under flow.**

**Y. Liu^1^, J. Crossen^1^, T. J. Stalker^2^, S. L. Diamond^1^***

1 Department of Chemical and Biomolecular Engineering

Institute for Medicine and Engineering

University of Pennsylvania

Philadelphia, PA 19104 USA

2 Department of Medicine

The Cardeza Foundation for Hematologic Research

Thomas Jefferson University, Philadelphia, PA

*Corresponding Author:

Scott L. Diamond, PhD

Department of Chemical and Biomolecular Engineering

Institute for Medicine and Engineering

1024 Vagelos Research Laboratory

University of Pennsylvania

Philadelphia, PA 19104, USA.

Tel: 215-573-5702

fax: 215-573-7227

email: sld@seas.upenn.edu

**Supplemental Movies S1-S2**

Supplemental Movie 1.

**Supplemental Movie S1. Confocal movie in z-direction for clots with α2-AP peptide.** HCTI whole blood monolayer were formed at venous shear (100 s^-1^) for 90 seconds with platelet and fibrin label then washed with buffer added α2-AP peptide for 2 mins. Confocal movie in z-direction labeled with platelet (red), fibrin (green) and α2-APF (blue).

Supplemental Movie 2.

**Supplemental Movie S2. α2-APF colocalizes with fibrin during thrombus formation in vivo.** Representative movie of thrombi 3.5 minutes after laser-induced vascular injury of mouse cremaster arterioles. Mice were infused with α2-antiplasmin fluorescent peptide. Platelets were labeled in red, fibrin were labeled in blue, and α2-AP is in green.

**Supplemental Figures S1-S5**

Supplemental Figure 1.


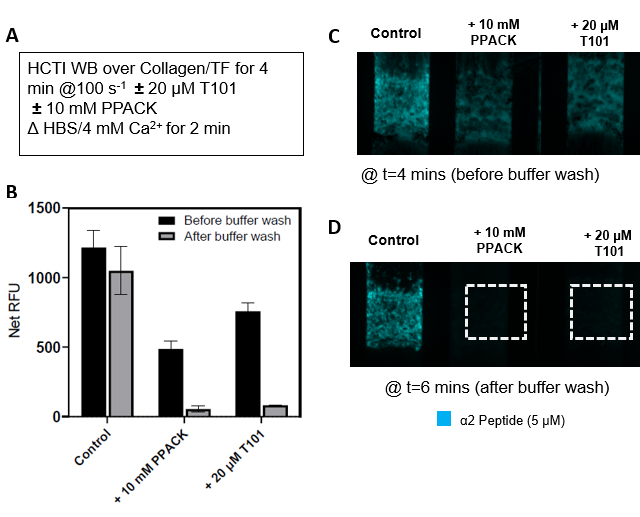


**Supplemental Figure S1. α2-APF FI before and after buffer wash with/without PPACK or T101.** (A)HCTI whole blood clots were formed at venous shear (100 s^-1^) for 240 seconds (4 minutes) with ± 10 mM PPACK (no thrombin) or ± 20 µM T101 (no FXIIIa) then wash with Buffer and 4 mM Ca^2+^ for 2 mins to remove free thrombin and α2-APF. (B) α2-APF FI data before and after buffer wash for control condition, condition with PPACK and condition with T101. (C) Representative images of α2-APF before buffer wash for three conditions. (D) Representative images of α2-APF after buffer wash for three conditions.
